# Torque Teno Virus in Bronchoalveolar Lavage Fluid of Hematological Patients and Association With Pathogens

**DOI:** 10.1002/jmv.70942

**Published:** 2026-04-28

**Authors:** Mahdi Ouafi, Camille Cordier, David Beauvais, Serge Alfandari, Marie Titécat, Romain Dubois, Frédéric Wallyn, Séverine Loridant, Fanny Vuotto, Micha Srour, Céline Berton, Valérie Coiteux, Ibrahim Yakoub‐Agha, Boualem Sendid, Didier Hober, Enagnon Kazali Alidjinou

**Affiliations:** ^1^ Laboratoire de Virologie ULR3610, Univ Lille, CHU Lille Lille France; ^2^ Laboratoire de Parasitologie‐Mycologie, Centre de Biologie Pathologie Génétique, Centre Hospitalier Universitaire de Lille, Unité de Glycobiologie Structurale et Fonctionnelle (CNRS UMR 8576), INSERM U1285, Université de Lille Lille France; ^3^ CHU de Lille, Maladies du Sang, Université de Lille Lille France; ^4^ Service Universitaire de Maladies Infectieuses et Tropicales, Hôpital Gustave Dron Tourcoing France; ^5^ Laboratoire de Bactériologie, CHU Lille, Université de Lille, U1286 INFINITE, Institut de recherche translationnelle sur l'inflammation Lille France; ^6^ Département de Pathologie CHU de Lille Lille France; ^7^ Institut Cœur‐Poumon, CHU Lille Lille France; ^8^ Service de Maladies Infectieuses, CHU de Lille Lille France

**Keywords:** bronchoalveolar lavage fluid, hematology, lower respiratory tract infection, Torque teno virus

## Abstract

Torque teno virus (TTV) replication is tightly modulated by the host immune response. As such, TTV viremia has been investigated as a biomarker of immune competence for monitoring the risks of allograft rejection and opportunistic infections in solid organ transplant recipients. We aimed to investigate the associations between TTV levels in bronchoalveolar lavage fluid samples (BALF) and pathogen detection in patients with hematological diseases with suspicion of lower respiratory tract infection (LRTI). TTV DNA was quantified in BALF using qPCR. Linear and logistic regressions were used to study associations between TTV levels in BALF and patients' characteristics, cytological patterns and pathogen detection. A total of 330 BALF from 272 patients with hematological malignancies were included. TTV DNA was detected in 44.5% of samples. The median TTV viral load (VL) in positive samples was 6.24 log copies/mL (IQR: 4.68–7.66). Strong positive associations were found between TTV levels in BALF and a diagnosis of lymphoma, a neutrophil fraction > 3% of BALF cells, and the detection of at least one respiratory virus. To a lesser extent, allogeneic hematopoietic stem cell transplantation, significant bacterial growth in BALF culture, and cytomegalovirus detection in BALF were also associated with TTV levels. We identified in this study multiple associations with TTV VL in BALF. These findings suggest that high TTV levels in BALF could reflect an impaired local immune status, and correlate with the risk of viral LRTI in immunocompromised patients.

## 1 Introduction

In 1997, Nishizawa et al. [1] identified a novel virus in a patient with post‐transfusion hepatitis. The virus was initially designated TT virus after the initials of the index patient. This virus was renamed as Transfusion‐Transmitted virus, after subsequent detection in blood donors and a variety of blood‐derived products [2]. Later, in reference to its circular, necklace‐shaped genome, it was finally termed Torque Teno Virus (TTV). TTV is the first recognized member of the family *Anelloviridae*, which encompasses numerous circular single‐stranded (ss) DNA viruses that infect a wide range of animal species, including humans.

Among the 37 currently established genera within this family, those found to infect humans include *Alphatorquevirus* (TTV species), *Betatorquevirus* (Torque Teno mini Virus species), *Gammatorquevirus* (Torque Teno midi Virus species), and recently *Gyrovirus* [3]. Anelloviruses are widely distributed in human populations worldwide, and are considered as part of the human virome. TTV were found to be the most abundant and received the greatest interest [4].

TTV has been detected in almost all types of human specimens, with reported prevalence varying widely depending on methodological sensitivity (PCR [polymerase chain reaction] *vs*. sequencing approaches, and primer sets) and geographic location. To date, no direct pathogenic effect has been demonstrated for these viruses, and they are currently considered commensals [5]. Their replication, however, is tightly modulated by host immune responses; it is estimated that approximately 90% of the 10^10^ virions per milliliter produced in the human body are cleared by immune mechanisms [6, 7]. Moreover, circulating viral load (VL) has been correlated with the degree of immunosuppression, particularly reflecting T‐cell function [8]. This has prompted investigation into the use of TTV viremia as a biomarker for monitoring immune competence in transplant recipients, especially kidney graft patients, to optimize immunosuppressive therapy and reduce risks associated with underexposure (allograft rejection) or overexposure (opportunistic infections) [9, 10]. Thresholds for plasmatic TTV VL to guide clinical decision‐making and follow‐up have been reported [11, 12].

Similarly, patients with hematological diseases are at a high risk of opportunistic infections [13] due to both disease‐related immunosuppression and the effects of various treatments. This risk is particularly pronounced in recipients of allogeneic hematopoietic stem cell transplantation (HSCT), owing to the effects of conditioning regimens and immunosuppressive therapies used to prevent graft‐versus‐host disease (GVHD).

Several studies have investigated the use of TTV VL in blood as an immune status biomarker in hematological patients, and have reported conflicting results regarding the association with opportunistic infections and immune‐related clinical events, mainly due to heterogeneity in patient characteristics, study design and PCR assays [14, 15, 16, 17, 18].

Lower respiratory tract infections (LRTI) are among the most common opportunistic infections in patients with hematological diseases and represent a major cause of morbidity and mortality [19, 20, 21]. Host factors and the specific types of associated immunodeficiencies mainly determine the pathogens involved. These factors, together with clinical and microbiological criteria, are often necessary for a comprehensive etiological diagnosis of LRTI in immunocompromised patients [22]. We hypothesized that TTV levels in bronchoalveolar lavage fluid (BALF) could reflect the local immune microenvironment, providing relevant information in immunocompromised patients with suspected LRTI.

TTV detection in BALF samples has been reported in few metagenomic studies, especially in lung transplant recipients [23, 24]. TTV levels in BALF have been recently investigated in a small cohort of various immunocompromised patients and were suggested to be a potential predictor for opportunistic infections [25].

We aimed to investigate TTV VL in BALF samples of patients with active hematological diseases with a suspicion of LRTI, and its association with the detection of pathogens.

## 2 Materials and Methods

### 2.1 Patients and Samples

We retrospectively included all patients admitted to the hematology department between January 2021 and July 2024, who underwent BALF sampling and microbiological investigations for suspicion of LRTI, and with available sample stored at −80°C. Only the first BALF after admission was included. In case of multiple hospital admissions during the study period for the same patient, independent inclusions were considered if there was a period of at least 30 days between samples.

Patients without active hematological disease or ongoing treatment were excluded.

Clinical and routine laboratory data were retrospectively retrieved from electronic medical records.

### 2.2 BALF Sample Collection

BAL were performed using flexible bronchoscopy under local anesthesia with lidocaine or under general anesthesia, depending on the clinical context. The bronchoscope was advanced distally into a lung region showing abnormalities on computed tomography.

Up to a maximum of 250 mL of sterile saline was instilled into the target area using 50‐mL syringes. Lavage fluid was recovered by gentle aspiration at −60 to −80 cmH₂O.

The first instilled syringe was considered bronchial wash and was not included in BAL analysis.

### 2.3 Microbiological and Cytological Investigations in BALF

BALF samples from immunocompromised patients, sent to the microbiology department for suspicion of LRTI, underwent standardized microbiological diagnostic procedures and assays for the detection of pathogens.

Microscopic examination and culture were performed to identify classical bacteria in BALF. Bacterial growth was considered significant when ≥ 10^4^ CFU/mL [26], or any monoculture of a bacterial pathogen was present. Mycobacteria were specifically tested with microscopy and culture.

Fungi were investigated in BALF through microscopy, culture, and specific qPCR (quantitative PCR) for *Aspergillus fumigatus* and *Pneumocystis jirovecii*. In addition, galactomannan antigen (Platelia *Aspergillus* Ag, Bio‐Rad, Hercules, United States) was tested in both BALF and serum samples. Mycological EORTC/MSGERC consensus criteria were used for the diagnosis of invasive aspergillosis [27]. Several RT‐qPCR and qPCR assays were used to investigate classical respiratory viruses and herpesviruses, namely AltoStar CMV/EBV/HSV/HHV‐6/VZV PCR Kit 1.5 (Altona diagnostics, Hamburg, Germany), Allplex Respiratory Panel 1, 2, and 3 (Seegene, Seoul, South Korea) which detects Influenza A and B viruses, Respiratory Syncytial Virus A and B, Adenovirus, Enterovirus, Metapneumovirus, Parainfluenza virus 1, 2, 3, and 4, Bocavirus, Coronavirus 229E, NL63 and OC43, and Human Rhinovirus, as well as the Simplexa COVID‐19 Direct Kit (Diasorin, Saluggia, Italy). Proportions of BALF cellular components were analyzed using abnormal cut‐off values based on the literature and the American Thoracic Society guidelines, which report normal values of healthy, nonsmoking adults [28, 29].

### 2.4 Quantification of TTV DNA in BALF

All BALF samples undergoing mycological testing were stored at −80°C when sufficient volume was available. Briefly, samples were centrifuged for 10 min at 3800*g*. Supernatants were removed to keep a 500 µL cellular fraction, then submitted to bead‐beating by 425 µm glass‐beads with Roche MagNA Lyser Instrument (Roche Diagnostics GmbH, Mannheim, Germany) for 70 s at 7000 rotations. DNA was extracted and eluted into 150 µL using the NucleoSpin Tissue kit (Macherey‐Nagel GmbH, Düren, Germany) on the HAMILTON Microlab STARlet system (Hamilton Company, NV, USA).

TTV levels were determined using an in‐house duplex qPCR using primers and probe previously reported to target TTV [30, 31] and albumin as housekeeping control gene [32]. The pan‐TTV assay targets and amplifies a 63 nucleotides fragment of the highly conserved untranslated region of the TTV genome. The primer and probe sequences employed were as follows: forward primer GTGCCGIAGGTGAGTTTA (position 177–194), reverse primer AGCCCGGCCAGTCC (position 226–239), and probe FAM‐TCAAGGGGCAATTCGGGCT‐BHQ1 (position 205–223). The amplification was performed using the Taqman Universal Master Mix, in a final volume of 25 μL containing 5 μL of extracted TTV DNA, on a QuantStudio5 cycler (Thermofisher, Villebon‐sur‐Yvette, France). The final concentrations for forward primer, reverse primer and probe were 400, 400, and 100 nM for TTV, and 200, 200, and 100 nM for albumin gene. The cycling conditions were: polymerase activation at 95°C for 15 min; 45 cycles of 95°C for 10 s and 60°C for 40 s. Dilutions of a synthetic TTV sequence (Eurofins, Luxembourg City, Luxembourg) previously quantified with the commercial TTV‐RGENE assay (Biomérieux, Marcy‐l'Etoile, France) were used to generate the standard curve. The limit of quantification of the assay was set at 1 copy/µL of eluate (corresponding 2.70 log copies/mL). This in‐house assay displayed an excellent correlation with the commercial TTV‐RGENE assay. TTV PCR results were interpreted as follows: (i) positive: TTV target detected; (ii) negative: TTV target not detected and albumin target detected; (iii) uninterpretable and sample retesting required: none of the targets detected.

### 2.5 Statistical Analysis

Quantitative data were presented as median and interquartile range (IQR) and categorical data were expressed as percentage. Linear and logistic regression models were used to investigate associations with TTV levels. Multivariable analyses included variables with *p*‐values < 0.10 from univariable analysis, which were corrected for multiple testing using the Benjamini–Hochberg procedure, as well as pre‐defined clinically relevant variables (age, sex, HSCT status, and BALF total cell count): variables with more than 20% missing values were excluded; variables exhibiting multicollinearity were excluded by retaining only the variable most strongly associated with the outcome; samples with missing data in any selected variable were also excluded; no data imputation was performed. Associations between variables and outcomes were expressed as adjusted regression coefficients or adjusted odds ratios with corresponding 95% confidence intervals.

For the purpose of linear regression, all TTV VL results below the limit of quantification were imputed at that limit. A *p*‐value < 0.05 was considered significant. All statistics and figures were produced using R program version 4.5.1.

### 2.6 Ethics

This study was conducted in compliance with the French reference methodology MR‐004, with approval of the local data protection committee under the number DEC25‐126. Non‐opposition consent was obtained from each patient included.

## 3 Results

### 3.1 Patient Characteristics

A total of 330 BALF from 272 patients were included. The characteristics of patients are summarized in Table 1. Briefly the median age was 60 years and males accounted for 63.2% of the patients. Acute leukemia (40.4%) and lymphoma (32.7%) were the most common diseases. Nearly half of the patients (47.1%) had undergone at least one allogeneic HSCT prior to BALF sampling. Of the 272 patients, 223 contributed a single BALF, 40 contributed two, and nine contributed three. The median interval between two BALF was 122 days. A detailed description of the cohort is provided in Supporting Information S1: Table S1.

**Table 1:** Demographic and clinical features.

Patients' characteristics, *N* = 272		
Age (years), median (IQR)		60 (49.75–68)
Male, *n* (%)		172 (63.24)
Main diagnosis, *n* (%)	Acute leukemia	111 (40.81)
	Lymphoma	89 (32.72)
	Myelodysplastic syndrome	20 (7.35)
	Plasma cell neoplasm	19 (6.99)
	Other hematological diseases	33 (12.13)
Allogeneic hematopoietic stem cell transplant recipient, *n* (%)		128 (47.06)
Time in days between BALF samples of the same patient, median (IQR)		122 (61–267)

*Note:* N: number.

Abbreviation: BALF, bronchoalveolar lavage fluid.

## 4 Results of Laboratory Investigations in BALF

The evaluation of cellular patterns and microbiological testing were performed for routine care. The results are presented in Table 2.

**Table 2:** Distribution and descriptive statistics of studied cytological and microbiological variables in BALF.

Category	Variable	Sample size	*N* (%)	Median [IQR]
BALF differential cell counts	Total cell count (cells/mm^3^)	301		210.00 [120.00–420.00]
	Macrophage fraction < 86%	307	217 (70.7)	
	Macrophage count (cells/mm^3^)	300		138.20 [72.30–241.10]
	Lymphocyte fraction > 15%	307	107 (34.9)	
	Lymphocyte count (cells/mm^3^)	300		22.60 [7.75–59.17]
	Neutrophil fraction > 3%	307	190 (61.9)	
	Neutrophil count (cells/mm^3^)	300		12.55 [2.10–60.75]
Bacteria in BALF	Significant bacterial growth	327	48 (14.7)	
	Isolation of Gram‐negative bacteria among those with significant bacterial growth	48	35 (72.9)	
	Isolation of mycobacteria	323	2 (0.6)	
Fungi in BALF	Presence of EORTC/MSGERC consensus mycological criteria for invasive aspergillosis	330	62 (18.8)	
	Positive microscopical examination for mold	330	5 (1.5)	
	Positive culture for *Aspergillus spp*.	330	17 (5.2)	
	Positive qPCR for *Aspergillus fumigatus*	330	51 (15.5)	
	*Aspergillus fumigatus* load (Ct)	51		35.98 [31.12–38.24]
	Galactomannan antigen index ≥ 0.5 in BALF	330	23 (7.0)	
	Galactomannan antigen index ≥ 0.5 in serum	330	14 (4.2)	
	Positive direct examination for *Pneumocystis jirovecii*	330	4 (1.2)	
	Positive qPCR for *Pneumocystis jirovecii*	330	39 (11.8)	
	*Pneumocystis jirovecii* load (Ct)	39		36.60 [33.00–39.50]
Pathogenic viruses in BALF	Detection of at least one *Orthoherpesviridae* virus	325	104 (32.0)	
	Detection of Cytomegalovirus	325	26 (8.0)	
	Cytomegalovirus load (log IU/mL)	26		2.88 [2.22–3.74]
	Detection of Epstein‐Barr virus	324	41 (12.7)	
	Epstein‐Barr virus load (log IU/mL)	41		2.44 [2.21–2.93]
	Detection of Herpes simplex virus 1	323	20 (6.2)	
	Herpes simplex virus 1 load (Ct)	20		32.70 [27.86–36.19]
	Detection of human herpes virus‐6B	321	55 (17.1)	
	Human herpes virus‐6B load (log IU/mL)	55		1.95 [1.72–2.59]
	Detection of at least one respiratory virus	326	86 (26.4)	
	SARS‐CoV‐2 load (Ct)	24		23.35 [18.68–31.25]
	Non‐SARS‐CoV‐2 respiratory viruses load (Ct)	64		27.55 [23.78–32.33]
	Detection of SARS‐CoV‐2	93	24 (25.8)	
	Detection of Rhinovirus	324	25 (7.7)	
	Detection of Parainfluenza viruses	324	16 (4.9)	
	Detection of human coronaviruses	324	10 (3.1)	
	Detection of Influenza A	324	8 (2.5)	
	Detection of other respiratory viruses	324	10 (3.1)	
Torque Teno Virus in BALF	Detection of TTV	330	147 (44.5)	
	TTV load above quantification threshold	330	136 (41.2)	
	TTV load among those above the quantification threshold (log copies/mL)	136		6.24 [4.68–7.66]

*Note:* N: number of patients. Other respiratory viruses: Influenza B, respiratory syncytial virus A and B, Adenovirus, Enterovirus, Metapneumovirus and Bocavirus.

Abbreviations: BALF, bronchoalveolar lavage fluid samples; Ct, quantitative polymerase chain reaction cycle threshold value; IQR, interquartile range; IU, international unit; SARS‐CoV‐2, severe acute respiratory syndrome coronavirus 2; TTV, torque teno virus.

The median total cell count in BALF was 210 [IQR: 120–420] cells/mm^3^. Macrophage, lymphocyte and neutrophil counts were 138 [IQR: 72–241], 23 [IQR: 8–59], and 13 [IQR: 2–61] cells/mm^3^, respectively. The cellular composition of BALF was studied as a reflection of the immune cellular response to infections, with defined abnormal proportion cut‐offs for macrophages (< 86%), lymphocytes (> 15%) and neutrophils (> 3%) [28, 29]. In our cohort, abnormal proportions for macrophages, lymphocytes and neutrophils accounted for 70.7%, 34.9%, and 61.9% of samples, respectively.

Bacterial culture was performed for 327 of the 330 BALF samples. A significant bacterial growth in BALF was recorded in 48 samples (14.7%). Mycobacterial culture was performed for 323 of the 330 BALF samples and a mycobacterial infection was detected in two samples from a single patient.

Testing for fungal infections was performed for all samples. A positive culture for *Aspergillus spp*. and a molecular detection for *Aspergillus fumigatus* were recorded in 17 samples (5.2%) and 51 samples (15.5%), respectively. Galactomannan antigen was positive in 23 BALF samples (7.0%). Overall, mycological EORTC/MSGERC consensus criteria were met for 62 samples (18.8%). *Pneumocystis jirovecii* was detected with specific qPCR in 39 samples (11.8%).

Regarding viral infections, classical respiratory viruses were tested in 326 samples, except for SARS‐CoV‐2, which was tested in only 93 samples. At least one respiratory virus was recorded in 86 samples (26.4%). In addition, orthoherpesviruses were investigated in 325 BALF samples, with a total of 104 samples (32.0%) found positive for at least one virus.

TTV quantification was retrospectively performed on stored samples. TTV DNA was detected in 147 BALF (44.5%), with levels above the TTV quantification threshold in most samples (136, 92.5%). In these samples, the median TTV VL was 6.24 log copies/mL [IQR: 4.68–7.66]. The TTV VL was above 6 log copies/mL in 75 samples (22.7%).

### 4.1 Factors Associated With TTV VL Levels

Linear regression was performed to investigate associations with TTV levels. The full set of univariable analyses for all studied variables is presented in the Supporting Information S2: Table S2.

Furthermore, in samples with quantifiable TTV DNA, correlations were sought between TTV levels and both BALF cellular component counts (Supporting Information S5: Figure S1A–D) and detected pathogen loads (Supporting Information S6: Figure S2A–H). A positive correlation was observed with the total number of cells, as well as the counts of different components. Regarding pathogens, it is noteworthy that in the 12 BALF samples positive for both TTV and *P. jirovecii*, a negative correlation was observed between Ct (cycle threshold) values (Spearman's ρ = −0.67, *p* = 0.017, Supporting Information S2: Figure S2B).

Among the 294 samples with a complete dataset, a multivariable linear regression analysis was carried out to identify factors independently associated with TTV VL levels. The results are presented in Table 3.

**Table 3:** Factors associated with TTV levels in BALF (log copies/mL): Linear regression univariable and multivariable analysis.

Variables	Univariable		Multivariable (*N* = 294)*	
	*β* [95% CI]	pFDR	aβ [95% CI]	*p*‐value
Age in years (*N* = 330)	0.01 [−0.00–0.03]	0.2034	0.01 [−0.01–0.02]	0.2361
Male sex (*N* = 330)	0.19 [−0.28–0.66]	0.5470	0.14 [−0.29–0.58]	0.5212
Allogeneic HSCT (*N* = 330)	−0.21 [−0.66–0.24]	0.4891	0.48 [0.00–0.95]	0.0487**
Lymphoma (*N* = 330)	1.20 [0.73–1.66]	< 0.0001	1.16 [0.66–1.67]	< 0.0001**
BALF total cell count in cells/mm^3^ (*N* = 301)	0.00 [−0.00–0.00]	0.0329	0.00 [−0.00–0.00]	0.7934
BALF neutrophil fraction > 3% (*N* = 307)	1.58 [1.13–2.04]	< 0.0001	1.21 [0.76–1.65]	< 0.0001**
Significant bacterial growth in BALF (*N* = 327)	0.92 [0.29–1.56]	0.0294	0.67 [0.06–1.29]	0.0320**
Detection of cytomegalovirus in BALF (*N* = 325)	1.35 [0.52–2.18]	0.0176	1.09 [0.30–1.89]	0.0073**
Detection of at least one respiratory virus in BALF (*N* = 326)	1.53 [1.04–2.02]	< 0.0001	1.15 [0.66–1.63]	< 0.0001**

*Note:* Respiratory virus detected include: Severe acute respiratory syndrome coronavirus 2, influenza A and B viruses, respiratory syncytial virus A and B, Adenovirus, Enterovirus, Metapneumovirus, Parainfluenza virus 1, 2, 3, and 4, Bocavirus, Coronavirus 229E, NL63 and OC43 and Human Rhinovirus; *: Only samples with a complete dataset across variables were included in the multivariable model; **: Variables reaching statistical significance for independent association with TTV levels in the multivariable analysis

Abbreviations: aβ, adjusted linear regression coefficient; BALF, bronchoalveolar lavage fluid; CI, confidence interval; HSCT, hematopoietic stem cell transplant recipient; pFDR, *p*‐value corrected using the Benjamini–Hochberg procedure, taking into account all tests performed (see Supporting Information S3: Table S3); qPCR, quantitative polymerase chain reaction; TTV, torque teno virus.

An increase in TTV levels was independently associated with patients with lymphoma (*β* = 1.16; 95% CI = [0.66–1.67]; *p* = < 0.0001), a neutrophil fraction > 3% of BALF cells (*β* = 1.21; 95% CI = [0.76–1.65]; *p* < 0.0001), detection of at least one respiratory virus in BALF (*β* = 1.15; 95% CI = [0.66–1.63]; *p* = < 0.0001), and to a lesser extent allogeneic HSCT (*β* = 0.48; 95% CI = [0.00–0.95]; *p* = 0.0487), significant bacterial growth in BALF (*β* = 0.67; 95% CI = [0.06–1.29]; *p* = 0.0320), and cytomegalovirus detection in BALF (*β* = 1.09; 95% CI = [0.30–1.89]; *p* = 0.0073).

The associations with lymphoma, a neutrophil fraction > 3% of BALF cells, detection of at least one respiratory virus in BALF and cytomegalovirus detection in BALF were also observed after normalization to the total cell number in BALF (see Supporting Information S3: Table S3).

### 4.2 Factors Associated With High TTV VL

To confirm the results obtained above, we investigated factors associated with high TTV levels. A threshold of 6 log copies/mL for high TTV VL has previously been used in blood as a marker of significant immunosuppression in kidney transplant recipients [11], and in nasal fluid from children with asthma, as a factor associated to impaired lung function [33]. We arbitrarily used for BALF this threshold, which also seemed relevant for TTV VL distribution in our cohort (22.7% of samples). Thereafter, samples with TTV VL above 6 log copies/mL are referred to as TTV‐high group (as compared to TTV‐low group).

Logistic regression was performed to investigate associations with high TTV levels. Detailed univariable analyses for all studied variables are presented in the Supporting Information S4: Table S4.

A diagnosis of lymphoma was more common in the TTV‐high group (54.7% vs. 25.5%).

Higher BALF median cell counts were observed in the TTV‐high group (370.00 cells/mm^3^ [182.50–605.00] vs. 190.00 cells/mm^3^ [100.00–320.00]). This increase was particularly pronounced for neutrophils, with higher median counts in the TTV‐high group (49.65 cells/mm^3^ [9.7–265.88] vs. 8.9 cells/mm^3^ [1.2–40.77]). The same trend was observed for the proportion of samples with an abnormal neutrophil count (84.5% vs. 55.1%).

Regarding pathogens, a detection of respiratory viruses was more common in in the TTV‐high group (49.3% vs. 19.5%).

Among the 297 samples with a complete dataset, a multivariable logistic regression analysis was performed to identify factors independently associated with a high TTV VL. The results are presented in Table 4.

**Table 4:** Factors associated with high TTV load in BALF (> 6 log copies/mL): Logistic regression univariable and multivariable analysis.

Variables	Univariable		Multivariable (*N* = 297)*	
	OR [95% CI]	pFDR	aOR [95% CI]	*p*‐value
Age in years (*N* = 330)	1.02 [1.00–1.03]	0.2594	1.02 [1.00–1.04]	0.1233
Male sex (*N* = 330)	1.33 [0.77–2.32]	0.5419	1.38 [0.72–2.64]	0.3284
Allogeneic HSCT (*N* = 330)	0.77 [0.46–1.29]	0.5507	2.03 [0.94–4.42]	0.0731
Lymphoma (*N* = 330)	3.52 [2.07–6.05]	< 0.0001	4.61 [2.16–9.80]	0.0001**
BALF total cell count in cells/mm^3^ (*N* = 301)	1.00 [1.00–1.00]	0.2594	1.00 [1.00–1.00]	0.7310
BALF neutrophil fraction > 3% (*N* = 307)	4.45 [2.31–9.32]	< 0.0001	3.57 [1.68–7.59]	0.0009**
Detection of at least one respiratory virus in BALF (*N* = 326)	4.01 [2.32–6.99]	< 0.0001	3.51 [1.89–6.53]	0.0001**

*Note:* Respiratory virus detected include: Severe acute respiratory syndrome coronavirus 2, influenza A and B viruses, respiratory syncytial virus A and B, Adenovirus, Enterovirus, Metapneumovirus, Parainfluenza virus 1, 2, 3, and 4, Bocavirus, Coronavirus 229E, NL63 and OC43 and Human Rhinovirus; *: Only samples with a complete dataset across variables were included in the multivariable model; **: Variables reaching statistical significance for independent association with high TTV load in the multivariable analysis.

Abbreviations: aβ, adjusted linear regression coefficient; BALF, bronchoalveolar lavage fluid; CI, confidence interval; HSCT, hematopoietic stem cell transplant recipient; pFDR, *p*‐value corrected using the Benjamini–Hochberg procedure, taking into account all tests performed (see Supporting Information S4: Table S4); qPCR, quantitative polymerase chain reaction; TTV, torque teno virus.

A TTV VL > 6 log copies/mL in BALF was independently associated with a diagnosis of lymphoma (aOR = 4.61; 95% CI = [2.16–9.80]; *p* = 0.0001), a neutrophil fraction > 3% of BALF cells (aOR = 3.57; 95% CI = [1.68–7.59]; *p* = 0.0009) and detection of at least one respiratory virus in BALF (aOR = 3.51; 95% CI = [1.89–6.53]; *p* = 0.0001).

## 5 Discussion

We investigated TTV levels in BALF as an assessment of local immune status in patients with hematological disease with suspicion of LRTI, and found strong positive associations with lymphoma, neutrophil counts in BALF and detection of respiratory viruses.

TTV was detected in 44.5% of samples. Our data agree with the report by Zhu et al. [25] in which TTV was detected in 37.1% of BALF from immunocompromised hosts versus 17.3% in non‐immunocompromised ones. In a cohort of hospitalized children, a TTV detection rate of 23% in sputum and BALF was recently reported [34]. In addition, high VL were frequent in our study, with TTV levels exceeding 6 log copies/mL in 22.7% of samples, a threshold assumed to reflect a significant local immunosuppression. This study provides evidence of different associations with TTV levels.

Lymphoma was independently associated with TTV levels in BALF. In a multivariable model, patients with lymphoma had 4.6‐fold higher odds of a high TTV VL as shown in Table 4. The association with lymphoid malignancies has been reported previously with TTV VL in plasma [15, 17]. Such findings may reflect the permissiveness of B tumoral cells to TTV replication, as previously demonstrated in Raji cells [35], a human B‐lymphoblastoid cell line. Most of lymphoma in our cohort were B‐cell lymphomas, and this could contribute to elevated TTV VL in BALF. Another host factor that appeared to be associated with TTV levels in our multivariable analysis model was allogenic HSCT, as presented in Table 3. The lack of association in univariable analysis is likely attributable to a suppressor effect [36], given that lymphoma, which is strongly associated with the outcome, was much less frequent among HSCT recipients (14.0% vs. 49.7%).

A noteworthy result of our study is a strong positive correlation between TTV levels and cellular richness of BALF samples, as outlined in Supporting Information S5: Figure S1. These correlations may simply reflect differences in dilution during BALF sampling, however, this effect was partially mitigated by the sample preparation procedure, which included a concentration step using centrifugation. In addition, a proportion of neutrophils above 3% of cells in BALF was associated with TTV levels as shown in Table 3. This neutrophil cut‐off is usually described as indicative of bacterial and fungal LRTI [29]. Interestingly, significant bacterial growth in BALF was linked to TTV levels, even if an association was not shown with high TTV VL, as reported in Supporting Information S2 and S4: Tables S2 and S4. This discrepancy may be due to the lower statistical power of the logistic regression model, resulting from the limited number of events with high VL. Recent studies found associations between TTV in BALF and both invasive aspergillosis and mycobacterial infection [25] and between TTV VL in nasopharyngeal specimens and bacterial infection [37]. Another possible explanation for an association with high neutrophil counts could be a neutrophil tropism for TTV, as recently suggested by its capacity to replicate in immature granulocytes [38, 39], however, this hypothesis deserves further investigation.

From a microbiological perspective, the most important finding of this study was a robust association between TTV levels and the detection of respiratory viruses in BALF. In a multivariable model, detection of respiratory viruses in BALF was associated with 3.5‐fold higher odds of high TTV VL, as presented in Table 4. Similar findings have been reported in nasopharyngeal swab samples [37, 40, 41], where elevated TTV levels were linked to poorer outcomes [37, 42]. Hypothetically, disruptions of physicochemical barriers, impaired ciliary movement, and local immune responses caused by respiratory viruses [43] may create a favorable environment for TTV replication. Moreover, elevated TTV levels in nasal fluid have been associated with lung dysfunction in children with asthma [33]. However, it remains unclear whether TTV merely takes advantage of these impairments, actively contributes to their severity, or could serve as a marker of such respiratory alterations.

Cytomegalovirus appeared to be the only herpes virus associated with TTV levels in BALF as outlined in Supporting Information S2: Table S2.

It's noticeable that even if no associations were observed with fungal agents, an inverse correlation was observed in a very small subgroup, between TTV and *P. jirovecii* loads as shown in Supporting Information S6: Figure S2. This specific point is currently under investigation.

Overall TTV levels in BALF appeared to be associated with specific patient characteristics, cytological patterns, and pathogen detection, suggesting that TTV VL might reflect both the local pulmonary immune microenvironment and the patterns of immunosuppression.

If these findings are confirmed and further explored, TTV VL in BALF could provide valuable insights into the complex interplay between immunosuppression and pathogen susceptibility. Indeed, such a tool along with others could help to draw an overview of pathogens which are more likely to infect patients based on their specific immune deficits, thereby refining individualized diagnostic and therapeutic strategies.

The primary limitation of this study was missing data points for some variables, due to its retrospective design. Radiological findings were not available, which limited the detailed diagnosis of LRTI, such as invasive aspergillosis, according to EORTC/MSGERC consensus criteria [27]. Clinical follow‐up was incompletely recorded, which precluded assessment of disease severity, symptom resolution, treatment response, attributable mortality, and hematological disease relapse. In addition, matched‐blood samples were unavailable, and therefore, blood TTV VL was not measured. Finally, the study lacked comparator groups, including immunocompetent individuals and other immunocompromised populations such as solid‐organ transplant recipients, patients with solid tumors, and non‐hematological patients with ventilator‐associated pneumonia in intensive care units.

A prospective study investigating TTV levels in BALF and blood from both immunocompromised and immunocompetent patients with LRTI will be of great interest and will refine the associations described in the current study.

While TTV VL has been studied extensively in blood and, more recently, in nasopharyngeal samples, this is the first study of this scale to assess TTV VL in BALF. Lower respiratory sampling remains essential for the diagnosis of most LRTI in immunocompromised patients and likely provides a better reflection of local alveolar immunity than blood or upper respiratory samples.

Our results suggest that TTV levels in BALF have the potential to serve as an assessment of local immune status and correlate with the risk of viral LRTI in immunocompromised patients.

## Author Contributions

Conceptualization: Mahdi Ouafi, Camille Cordier, Serge Alfandari, Boualem Sendid, and Enagnon Kazali Alidjinou. Data curation: Mahdi Ouafi, and Camille Cordier. Formal analysis: Mahdi Ouafi and Enagnon Kazali Alidjinou. Funding acquisition: Enagnon Kazali Alidjinou. Investigation: Mahdi Ouafi and Enagnon Kazali Alidjinou. Project administration: Mahdi Ouafi, Camille Cordier, and Enagnon Kazali Alidjinou. Resources: Mahdi Ouafi, Camille Cordier, David Beauvais, Serge Alfandari, Marie Titécat, Romain Dubois, Frédéric Wallyn, Séverine Loridant, Fanny Vuotto, Micha Srour, Céline Berton, Valérie Coiteux, Ibrahim Yakoub‐Agha, Boualem Sendid, Didier Hober, and Enagnon Kazali Alidjinou. Supervision: Camille Cordier and Enagnon Kazali Alidjinou. Validation: Mahdi Ouafi, and Enagnon Kazali Alidjinou. Visualization: Mahdi Ouafi. Writing – original draft preparation: Mahdi Ouafi, Camille Cordier, and Enagnon Kazali Alidjinou. Writing – review and editing: Mahdi Ouafi, Camille Cordier, David Beauvais, Serge Alfandari, Fanny Vuotto, Didier Hober, and Enagnon Kazali Alidjinou.

## Conflicts of Interest

The authors declare no conflicts of interest.

## Supporting information

Supporting Figure S1

Supporting Figure S2

Supporting Table S1

Supporting Table S2

Supporting Table S3

Supporting Table S4

## Data Availability

The data that support the findings of this study are available from the corresponding author upon reasonable request.
