# Supplementary material for: Torque Teno Virus in Bronchoalveolar Lavage Fluid of Hematological Patients and Association With Pathogens
Source: J Med Virol. 2026 Apr 28;98:e70942. doi: 10.1002/jmv.70942 (PMC13122748; doi:10.1002/jmv.70942)
Supplement: Supplementary file 1 — Supporting Figure S1 [file JMV-98-e70942-s002.pptx]

## Slide 1
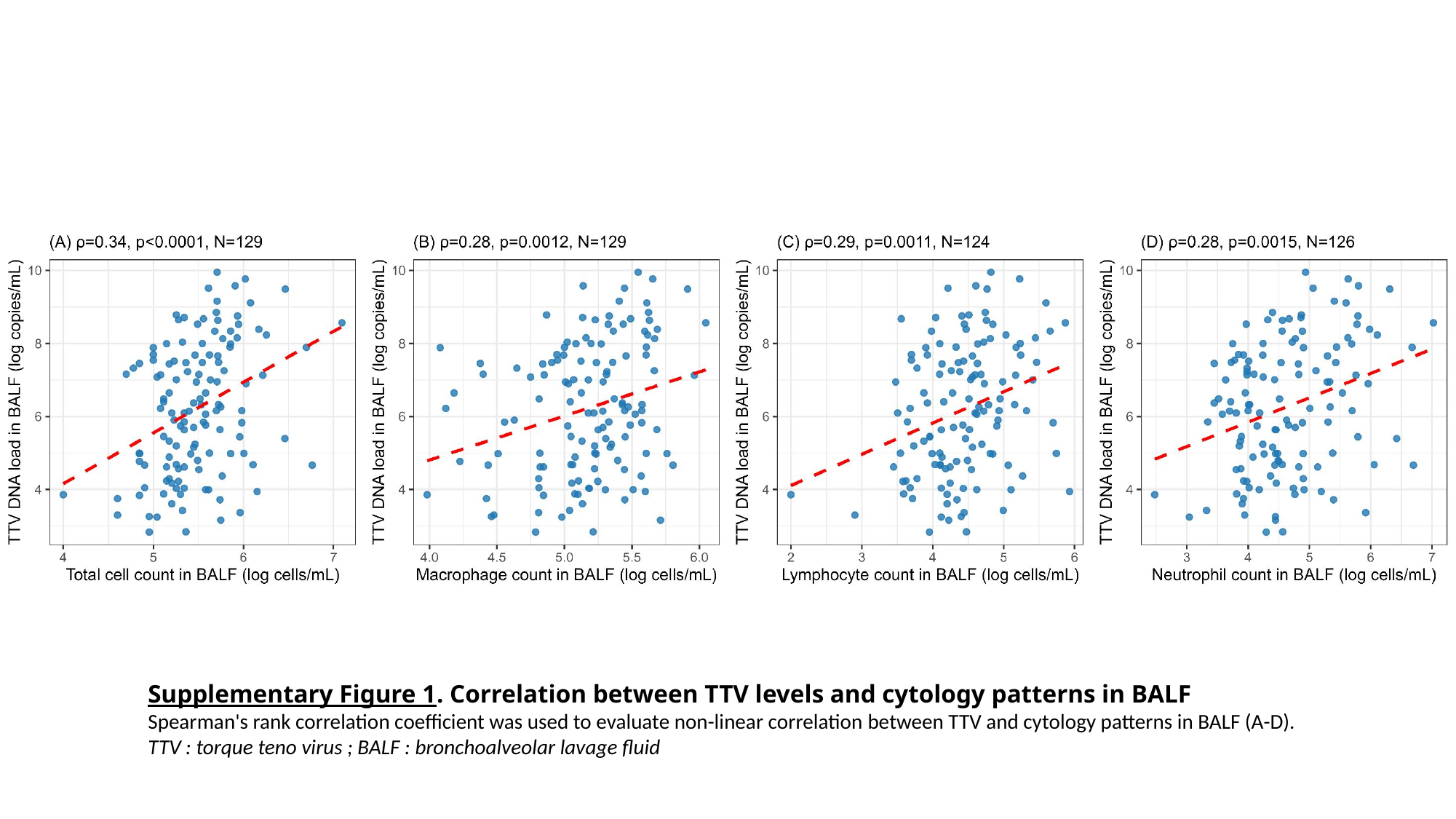

Supplementary Figure 1. Correlation between TTV levels and cytology patterns in BALF
Spearman's rank correlation coefficient was used to evaluate non-linear correlation between TTV and cytology patterns in BALF (A-D).
TTV : torque teno virus ; BALF : bronchoalveolar lavage fluid
