# Supplementary material for: Torque Teno Virus in Bronchoalveolar Lavage Fluid of Hematological Patients and Association With Pathogens
Source: J Med Virol. 2026 Apr 28;98:e70942. doi: 10.1002/jmv.70942 (PMC13122748; doi:10.1002/jmv.70942)
Supplement: Supplementary file 2 — Supporting Figure S2 [file JMV-98-e70942-s005.pptx]

## Slide 1
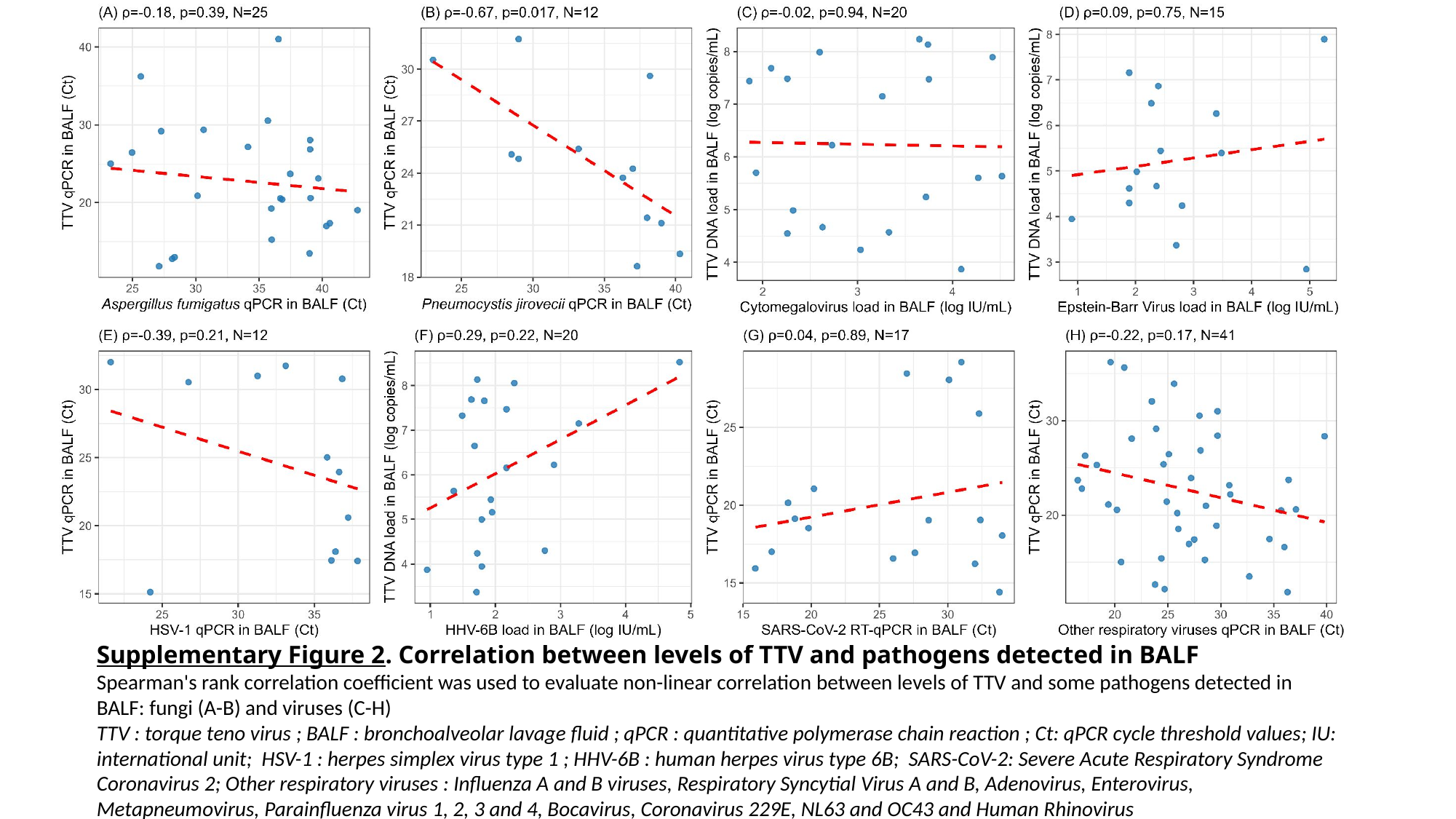

Supplementary Figure 2. Correlation between levels of TTV and pathogens detected in BALF
Spearman's rank correlation coefficient was used to evaluate non-linear correlation between levels of TTV and some pathogens detected in BALF: fungi (A-B) and viruses (C-H)
TTV : torque teno virus ; BALF : bronchoalveolar lavage fluid ; qPCR : quantitative polymerase chain reaction ; Ct: qPCR cycle threshold values; IU: international unit; HSV-1 : herpes simplex virus type 1 ; HHV-6B : human herpes virus type 6B; SARS-CoV-2: Severe Acute Respiratory Syndrome Coronavirus 2; Other respiratory viruses : Influenza A and B viruses, Respiratory Syncytial Virus A and B, Adenovirus, Enterovirus, Metapneumovirus, Parainfluenza virus 1, 2, 3 and 4, Bocavirus, Coronavirus 229E, NL63 and OC43 and Human Rhinovirus
