# Supplementary material for: Torque Teno Virus in Bronchoalveolar Lavage Fluid of Hematological Patients and Association With Pathogens
Source: J Med Virol. 2026 Apr 28;98:e70942. doi: 10.1002/jmv.70942 (PMC13122748; doi:10.1002/jmv.70942)
Supplement: Supplementary file 3 — Supporting Table S1 [file JMV-98-e70942-s004.docx]

**Supplementary Table 1. Detailed clinical features and treatments**

| **Clinical features and treatments, N=272** | | |
| --- | --- | --- |
| Main diagnosis, n (%) : | | |
| Acute leukemia | - Acute myeloid leukemia | 83 (30.5) |
|  | - Acute lymphoid leukemia | 27 (9.9) |
|  | - Acute biphenotypic leukemia | 1 (0.4) |
| \| Lymphoma \| - Hodgkin’s disease \| 13 (4.8) \| \| --- \| --- \| --- \| \| - B-cell non-Hodgkin’s lymphoma \| 62 (22.8) \| \| - T-cell lymphoma \| 14 (5.1) \| \| Myelodysplastic syndrome \| \| 20 (7.4) \| \| Plasma cell neoplasm \| - Multiple myeloma \| 17 (6.3) \| \| - Plasma cell leukemia \| 1 (0.4) \| \| - Waldenstrom’s disease \| 1 (0.4) \| \| Other types of hematological diseases \| - Primary myelofibrosis \| 6 (2.2) \| \| - Chronic lymphoid leukemia \| 5 (1.8) \| \| - Chronic myelomonocytic leukemia \| 5 (1.8) \| \| - Prolymphocytic leukemia \| 3 (1.1) \| \| - Secondary myelofibrosis \| 2 (0.7) \| \| - Primary immune deficiency disorders \| 2 (0.7) \| \| - Essential thrombocythaemia \| 2 (0.7) \| \| - Chronic myeloid leukemia \| 2 (0.7) \| \| - Anaplastic anemia \| 2 (0.7) \| \| - Microscopic polyangiitis \| 1 (0.4) \| \| - AL amyloidosis \| 1 (0.4) \| \| - Hemophagocytic lymphohistiocytosis \| 1 (0.4) \| \| - Unclassifiable myelodysplastic/ myeloproliferative disorder \| 1 (0.4) \| | | |
| HSCT recipient, n (%) | | 128 (47.1) |
| Treatments administered among HSCT recipients, n (%) : | | |
| HSCT graft from an unrelated donor | | 95 (74.2) |
| \| HSCT conditioning \| - Myeloablative conditioning \| 65 (50.8) \| \| --- \| --- \| --- \| \| - Reduced intensity conditioning \| 63 (49.2) \| | | |
| Total body irradiation | | 16 (12.5) |
| \| GVHD prophylaxis \| - Anti-lymphocyte globulins \| 69 (53.9) \| \| --- \| --- \| --- \| \| - Cyclosporine \| 127 (99.2) \| \| - Methotrexate \| 103 (80.5) \| \| - Cyclophosphamide \| 27 (21.1) \| \| - Mycophenolate mofetil \| 21 (16.4) \| \| - Alemtuzumab \| 1 (0.8) \| | | |
| Acute GVHD within 100 days post-HSCT | | 29 (22.7) |

*N: number of patients; BALF: HSCT: allogeneic hematopoietic stell cell transplantation; GVHD: graft versus host disease*
